# Deficits during Voluntary Selection in Adult Patients with ADHD: New Insights from Single-Trial Coupling of Simultaneous EEG/fMRI

**DOI:** 10.3389/fpsyt.2014.00041

**Published:** 2014-04-22

**Authors:** Susanne Karch, Julia Madeleine Voelker, Tobias Thalmeier, Matthias Ertl, Gregor Leicht, Oliver Pogarell, Christoph Mulert

**Affiliations:** ^1^Neurophysiology and Functional Neuroimaging, Department of Psychiatry and Psychotherapy, Ludwig-Maximilians-University, Munich, Germany; ^2^Psychiatry Neuroimaging Branch, Department of Psychiatry and Psychotherapy, University Medical Center Hamburg-Eppendorf, Hamburg, Germany

**Keywords:** ADHD, EEG, fMRI, executive functions, voluntary selection, event-related potentials

## Abstract

Deficits in executive functions, including voluntary decisions are among the core symptoms of attention deficit/hyperactivity disorder (ADHD) patients. In order to clarify the spatiotemporal characteristics of these deficits, a simultaneous EEG/functional MRI (fMRI) study was performed. Single-trial coupling was used to integrate temporal EEG information in the fMRI analyses and to correlate the trial by trial variation in the different event-related potential amplitudes with fMRI BOLD responses. The results demonstrated that during voluntary selection early electrophysiological responses (N2) were associated with responses in similar brain regions in healthy participants as well as in ADHD patients, e.g., in the medial-frontal cortex and the inferior parietal gyrus. However, ADHD patients presented significantly reduced N2-related BOLD responses compared to healthy controls especially in frontal areas. These results support the hypothesis that in ADHD patients executive deficits are accompanied by early dysfunctions, especially in frontal brain areas.

## Introduction

Numerous studies indicate deficits in patients with attention deficit/hyperactivity disorder (ADHD) in various cognitive abilities, including the inhibition of pre-potent responses and voluntary decisions (1–5). Dysfunctions of these executive functions like difficulties in cognitive control and in the ability to monitor and flexibly change and revise behavior in relation to goals or problems with focused attention are among the core symptoms in ADHD (2, 6, 7). The voluntary selection of response alternatives is related to neuronal responses in various brain areas, including lateral and medial-frontal areas (1, 5, 8–12) as well as parietal regions (9, 13, 14). Functional MRI (fMRI) studies have shown a fronto-striatal hypoperfusion (15–18) or decreased activity, e.g., in fronto-medial brain areas in ADHD patients during tasks with testing executive functions like the free selection of responses (18, 19). These results indicate altered brain functions in ADHD patients and may even be compensatory mechanisms with adaptively modulated cognitive processes (20). However, fMRI has a limited temporal resolution, which makes it difficult to disentangle the temporal dynamics of deficits in patients with ADHD.

By contrast, EEG analyses provide a more accurate temporal resolution. The N2, an event-related potential (ERP) which appears approximately 200 ms after a sensory stimulus, is known to be linked with early aspects of decision processes. The N2 has been associated with the first cognitive processing of a perceived stimulus (21), for example, stimulus identification and categorization processes (22), focusing of spatial attention or attentional shifts, suppression of surrounding non-target items (23, 24), and working memory maintenance (23, 25). Additionally, the N2 component has been described in association with conflict monitoring and detection of novelty or mismatch (26), stimulus identification (22), inhibition of motor responses, and overcoming stereotypical responses. Localization studies showed the N2 amplitude to be pre-dominantly located in medial-frontal areas, for example, in the anterior cingulate gyrus (ACC) (27–29).

The P3 has been linked to subsequent processing stages, which follow the response selection, like further signal processing, and also selective attention and working memory (21, 27, 30) as well as context updating processes, like updating one’s representation of the environment (24, 31–34).

Studies referring to voluntary selection processes demonstrated that frontal activations were linked to relatively early electrophysiological processes (N2 potential) whereas parietal brain regions corresponded pre-dominantly with later EEG potentials (e.g., P3 potential) (13, 35). In addition, different areas have been associated with different functions: the voluntary selection between tasks is attributed to early frontal potentials, whereas parieto-occipital activity has been associated with selection processes (1).

It is hypothesized that deficits regarding response selection and decision processes are linked to EEG abnormalities: for instance, previous EEG studies have shown delayed and reduced N2 amplitudes and P3 potentials in ADHD patients compared to healthy subjects (26, 36–38). These deficits probably indicate altered inhibitory and impulse control mechanisms. Other studies reported that the P3 potential is pre-dominantly altered in ADHD patients (39, 40). By contrast, a recent study provided some evidence that the N2 is pre-dominantly associated with altered behavior of ADHD patients (41). The authors emphasized the importance of replication studies in order to increase the reliability of their results (41).

Data concerning the exact temporal and spatial aspects of underlying dysfunctions in ADHD patients are lacking. In order to obtain a more precise analysis of the spatiotemporal characteristics of voluntary selection in ADHD patients, EEG and fMRI were acquired simultaneously for high temporal (EEG) (28, 42, 43) and spatial resolution (fMRI) (44–51). A previous simultaneous EEG–fMRI study on ADHD patients has demonstrated reduced frontal BOLD activity in patients during voluntary selection tasks whereas responses in the parietal cortex did not differ between groups (35).

The aim of the present study was to use more precise and recently developed techniques to directly integrate the analyses of EEG and fMRI in order to analyze temporal characteristics of selection-related responses in ADHD patients and healthy controls. The goal of the present reanalysis of the data (35) was to better understand and to clarify the deficits of ADHD patients in voluntary selection with single-trial coupling [see also Ref. (13, 46, 48, 52–54)]. The target was to have a close look at neuronal responses, which correlate specifically with relatively early (N2) and later (P3) electrophysiological processes during the voluntary decision between response alternatives, and to evaluate which aspects of stimulus processing are affected in patients with ADHD.

For this purpose, we used the single-trial estimation of N200 and P300 amplitudes and the correlation of the trial by trial variation of the electrophysiological responses with the respective fMRI BOLD responses. The integrating electrophysiological ERPs and fMRI data, based on blood flow differences, by direct coupling, results in a high temporal and spatial resolution of cognitive processes and related brain responses (55).

## Materials and Methods

### Subjects

Eight adults with ADHD (one woman, seven men; average age: 38.25 years; range: 26–47 years; IQ = 118.7 ± 9.66) and eight matched (sex, age, education, intelligence) healthy subjects (one woman, seven men; average age: 36.5 years; range: 24–49 years; IQ = 119.6 ± 7.84) without any neurologic/psychiatric diagnosis participated in a simultaneous EEG and fMRI analysis. All patients participating in the study were outpatients of the psychiatric clinic and were diagnosed with ADHD, assessed by: (1) meeting at least six of nine DMS-IV-criteria for hyperactivity and inattention or impulsivity in childhood, and at least five of nine DSM-IV-criteria for a diagnosis in adulthood. (2) Experiencing a moderate to severe level of impairment that can be attributed to ADHD symptoms. (3) Describing ADHD symptoms from childhood to adulthood in self-reports. The severity of the symptoms was measured by the Wender Utah Rating Scale (Wurs; self-report) and the Conners’ Adult ADHD Rating Scales (CAARS; self-report). Patients had to be free of any pharmacological drug treatment for at least 4 weeks and free of comorbid psychiatric and/or neurological diagnoses. All patients and healthy subjects had finished secondary school and were matched regarding years of education (patients mean = 15.5 ± 3.59; healthy subjects: mean = 15.9 ± 3.12; *p* > 0.05). Every subject taking part in present study had already participated in the former study, based on the same fMRI- and EEG-raw data, which was also used for the present investigation (35).

### Study design and paradigm

The experiment consisted of an adapted auditory go/no-go task [see also Ref. (35)]: during the *go condition* subjects were instructed to press a response button as quick as possible while minimizing errors; during the *no-go condition*, this response was to be inhibited. In the *voluntary selection condition*, participants were allowed to freely decide, whether to press the response button or not. The conditions were presented in pseudo-randomized order. In addition, neutral conditions were included as passive listening tasks. In all conditions, a combination of two tones were presented in intervals of 1000 ms, with a tone-duration of 50 ms and a pressure level of 100 dB via headphones (Resonance Technology, Inc., Van Mays, USA). After the presentation of the tones, an interval was included, during that the participants were instructed to respond according the conditions for 600 ms. After 600 ms, the fMRI measurement started for about 1000 ms. The fMRI measurements were followed by a break of about 300 ms break until the next trial started.

The particular combination of three different sinus tones (800, 1000, 1300 Hz) encoded the different conditions: all three active conditions, *go*, *no-go*, and the *voluntary selection task* started with a middle frequency tone (1000 Hz), whereas the passive control condition started with a 800-Hz tone. The *go condition* consisted of a combination of the middle frequency tone (100 Hz) and the high frequency tone (1300 Hz), the *no-go condition* comprised the combination of the middle frequency tone (100 Hz) and the low frequency tone (800 Hz) whereas the voluntary *selection condition* consisted of two middle frequency tones (1000 Hz). Each condition was presented 80 times, the *go condition* was presented 160 times. The intertrial interval was 3 s. Missed button presses during the *go condition* and button presses during the *no-go condition* were not included in the analysis. Regarding the voluntary task, the subjects were told that the ratio button press/no button press did not matter as long as it was in random order and approximately equally often. Additionally, subjects were asked not to alternate between the two options and not to count. Subjects who responded in each trial or did not respond at all during the voluntary selection task were excluded from the study, because they had not followed the study design. In general, the response rate was higher in ADHD patients. During the voluntary condition they pressed the button in 66.7% (SD 8.44), whereas the control group responded with button press in 54.4% (SD 12.23) of trials.

### Procedure

Prior to the acquisition of data, the participants were trained by receiving a practice block of at least 10 min in order to get used to the paradigm and understand the instructions. Then, the participants took part in the fMRI measurements for 25 min. The auditory stimuli were generated outside the MR environment using the BrainStim software package (Brain Products, Munich). Binaural sound transmission was performed using an air tubing sound delivery system [Resonance Technology, Inc., Van Mays, USA; see also Ref. (50)]. During the MRI measurements, the participants were asked to keep their right index finger on the button of the response box.

### Image acquisition and conventional analysis of MRI data

The BOLD responses were acquired during the execution of the cognitive task in a 1.5-T MRI (Siemens Sonata, T2*-weighted, TR = 3 s, TE = 53 ms; 10 slices; matrix 64 × 64; FoV 192 × 192; slices thickness: 8 and 0.4 mm interslice gap; gradient-echo EPI pulse sequence). Ten slices per person were recorded, parallel to the AC–PC line (line from the superior surface of the anterior commissure to the center of the posterior commissure) resulting in a voxel-size of 2.8 mm × 2.8 mm × 8.0 mm. Functional data were adapted on high-resolution anatomical data sets, which had been collected in advance using 3D T1-weighted sequences. Per person, 485 fMRI images were generated. The first five images were excluded because of unsatisfactory saturation effects.

The EEG/fMRI data acquisition occurred in temporal synchrony to the task. In order to reduce MR-provoked artifacts on the EEG data, an interleaved design was used. The MRI noise took about 1000 ms; the acquisition of MRI images started 700 ms after presentation of the auditory information. Therefore, the acquisition of BOLD acquisition and ERPs was done at different time points.

Further analyses were done with the BrainVoyager QX Version 2.4.2. 2070 and Version 4.9.6 (Brain Innovation, Maastricht, Netherlands). In order to avoid inhomogeneities of the magnetic field, the first five images at the beginning of each session were discarded. The pre-processing included a 3D motion correction, slice scan time correction, spatial smoothing with a full-width-half-maximum Gaussian filter (8.0 mm FWHM), and the alignment of the individual data to the Talairach brain. Statistical analysis was carried out using a general linear model approach. Each condition (voluntary selection, no-go, go, control) was modeled separately after convolution with a canonical hemodynamic response. Individual’s contrast images were used in the subsequent analysis in order to derive statistical maps. For group analysis, a second level fixed effects analysis (selection, no-go, go, control) was computed.

### EEG acquisition and pre-processing

EEG signals were recorded simultaneously without any filtering during acquisition. Recording was done with 61 Ag/AgCl electrodes placed on the scalp according to the international 10–10 system (reference: Cz; sampling rate: 5000 Hz; Brain Products, Munich), using an electrode cap set (Easycap, Germany). Impedances were usually maintained below 10 kΩ. An ECG was recorded with three electrodes placed on the participants’ back. One channel placed beneath the right eye was used to record eye movements. Concerning the post-processing, a 55-Hz low-pass filter (slope 24 dB/oct) was used. The MRI-artifact correction based on an averaging-algorithm was run in the timeframes of MRI acquisition (−50 ms until 1300 ms in relation to the start of the MRI acquisition; Vision Analyzer 2.0; Brain Products GmbH, Gilching, Germany). Cardio ballistic artifacts and eye movements were excluded by using the independent component analysis [EEGlab 6.01 based on Matlab 7.4 (Mathworks, Natick, MA, USA) (56)]. All channels are included in a matrix decomposition, which divided the EEG into components representing either brain activity or artifact (57). Afterward the corrected EEGs were obtained with the aid of the back-projection of the remaining independent components (55) and transferred to Vision Analyzer 2.0 (Brain Products, Gilching, Germany) for post-processing and final EEG data analyses. Further analysis included re-referencing to average reference and filtering with a 30-Hz low-pass filter, slope 24 dB/oct, and a 0.5305 high-pass filter [see also Ref. (58)]. The data were segmented according to the different experimental conditions (go, no-go, voluntary selection, control) in intervals of 300 ms before the stimulus until 600 ms after the stimulus presentation. A baseline correction was done based on the information of the 300-ms before stimulus presentation. After the baseline correction segments with signals exceeding voltage of ±90 μV/ms in central electrodes (Fz, Cz, FCz) were excluded. The segments were averaged separately for the different experimental conditions. Trials with incorrect responses (no response after go condition; button press after no-go or control condition) were also excluded prior to averaging.

### Single-trial coupling

**Figure 1:**
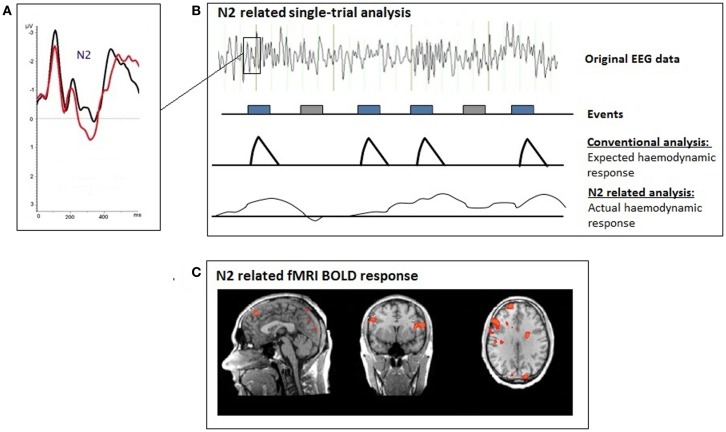
**Illustration of the single-trial coupling technique; (A) event-related potential (ERP); (B) the individual ERP amplitudes at each time-point after the presentation of the voluntary selection task were used for the single-trial GLM analyses; (C) single-trial N2-related fMRI response during voluntary selection between different response alternatives**.

A trial-by trial coupling of EEG and fMRI was used for the single-trial coupling after matrix decomposition of the specific N2 amplitude information (Fz) using Schmidt–Gram orthogonalization (see Figure 1). For the single-trial analysis, the N2 amplitude was measured at Fz (search window: 150–230 ms), the P3 amplitude at FCz (search window: 230–500 ms) separately for each stimulus presentation. Artifacts were defined as amplitudes exceeding the mean amplitude value by more than 2.5 SD. Amplitudes measured in artifact segments were set to the individual mean value. The N2- and P3-amplitudes were used for the calculation of the regressors (fMRI analysis).

A second level analysis fMRI was calculated: for that purpose the N2- and P3-amplitudes, which were measured separately for each trial were used. The function encoded the amplitude of the single-trial ERPs, measured at each frame in order to find brain regions whose responses were specific to the electrophysiological measure. ERP amplitudes were orthogonalized with respect to the target function (Schmidt–Gram orthogonalization) in order to detect hemodynamic responses specifically related to the electrophysiological response (amplitude of N2) and not to some general feature of the target detection process [see also Ref. (59, 55), for further details]. The orthogonalized ERP time series information was used to calculate regressors for N2- and P3-specific BOLD responses. The regressors were convolved with a hemodynamic response function and *z*-normalized before entering the GLM model. The GLM model included the regressors for N2- and P3-specific BOLD responses. A fixed effects analysis was done [corrected for multiple comparisons using the false detection rate *q*(FDR) < 0.01; *T* = (3.68–8); cluster >30 voxels] in order to compare the BOLD signal changes between the two groups.

**Figure 2:**
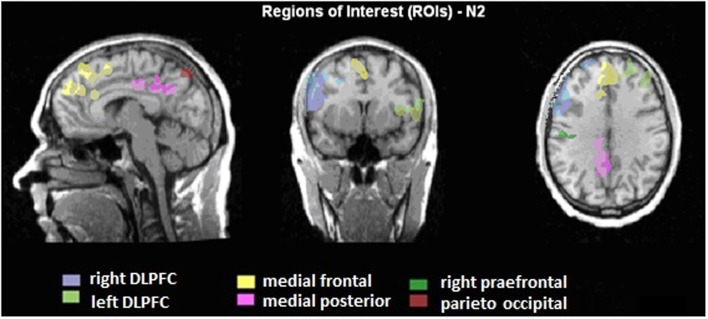
**Illustration of the regions of interest (ROIs) presented on talairachised anatomical image**. The ROIs are based on the BOLD activity, which is correlated to the N2 amplitudes during voluntary selection of healthy subjects.

A region of interest (ROI) analysis was carried out in order to directly compare the BOLD responses in different brain areas between the two groups. Six different regions were defined, based on the BOLD-activity of the healthy subjects during the voluntary selection task: DLPFC right and left, medial-frontal cortex, medial-posterior cortex, right pre-central gyrus, and parieto-occipital cortex (see Figure 2). For each subject, the average *T*-value of the activated voxels (*T*-score: 2.2–8; *p* < 0.05) was calculated. Afterward, the number of activated voxels in the different ROIs between ADHD patients and healthy subjects was compared with the Mann–Whitney *U* test for independent groups.

### ERP Statistics

Statistics were calculated using the program SPSS Version Statistics 19. The significance level was *p* < 0.05. Differences regarding the N2 amplitudes were assessed using a multivariate analysis for repeated measurements (MANOVAs) with the factors electrode position (Fz, FCz, Cz) and task (go, no-go, voluntary selection). Additionally, the intersubject factor ADHD patient vs. healthy subject was calculated. In case of a significant Mauchly test of sphericity, a Greenhouse-Geisser correction was used. In addition, *post hoc t*-tests were used (Bonferroni-correction).

## Results

### ERP results

**Figure 3:**
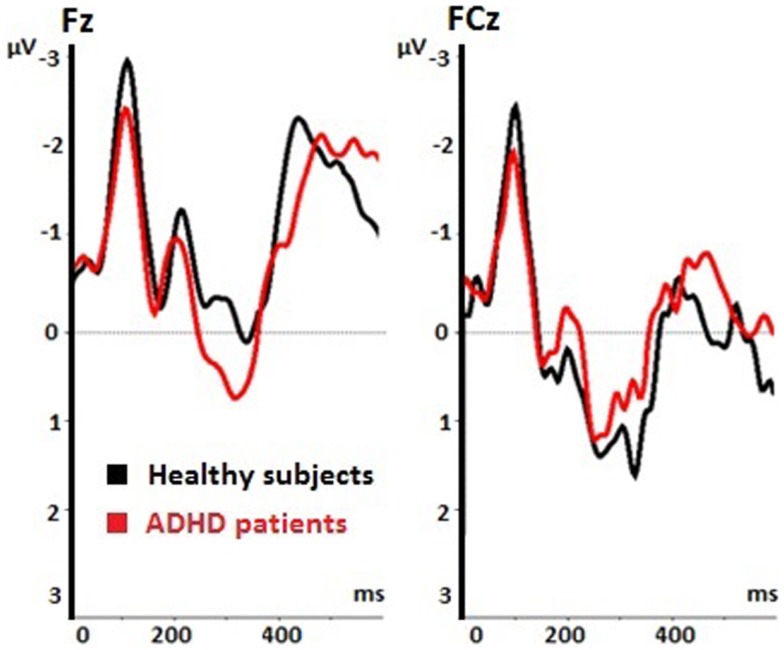
**Event-related potentials (ERPs) during voluntary selection condition in fronto-central electrodes (Fz, FCz) of healthy controls (black) and ADHD patients (red); μV, microvolt; ms, milliseconds**.

The voluntary selection task led to an early negative ERP (N2) in both groups, especially in fronto-central areas. Regarding the N2 amplitude, we found a significant main effect of electrode position [*F*(1.149, 16.085) = 14.918; *p* < 0.001]. The *post hoc* comparisons revealed significant different amplitudes between Fz and FCz (*p* = 0.001): the N2 amplitude in Fz was increased compared to FCz (see Table 1). The main effect of task [*F*(2, 28) = 2.601; *p* = 0.092] and the interaction effect (task × electrode) [*F*(4, 56) = 2.342; *p* = 0.066] were not significant. The N2 amplitudes did not differ between groups [*F*(1, 14) = 0.58; *p* = 0.813] (see Figure 3). Interaction effects (task × group) [*F*(2, 28) = 0.176; *p* = 0.839] (electrode × group) [*F*(1.149, 16.085) = 0.104; *p* = 0.786] and (task × electrode × group) [*F*(4, 56) = 2.342; *p* = 0.786] were not significant either.

**Table 1:** **N2 amplitudes during voluntary selection measured at different central electrodes**.

	Healthy subjects		ADHD patients	
	N2 amplitude/ μV (mean value)	SD	N2 amplitude/ μV (mean value)	SD
Volition
Fz	−2.718	±3.487	−1.526	±2.117
FCz	−1.157	±1.975	−1.067	±1.168

Patients and healthy controls showed a P300 potential during voluntary selection tasks, especially in FCz. The P3 was decreased in Fz than fronto-centrally (*p* = 0.001) (see Table 2). Although the P3 amplitude of healthy participants was 1.365 μV higher than the P3 amplitude of ADHD patients, the difference was not significant [*F*(1, 14) = 1.656; *p* = 0.219] (see Table 2).

**Table 2:** **P3 amplitudes during voluntary selection measured at different central electrodes**.

	Healthy subjects		ADHD patients	
	P3 amplitude/ μV (mean value)	SD	P3 amplitude/ μV (mean value)	SD
Volition
Fz	2.854	±4.767	1.681	±2.865
FCz	4.718	±5.020	2.545	±2.284

### Functional MRI results

**Figure 4:**
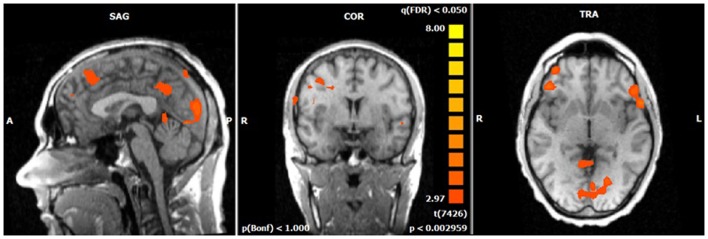
**N2-related BOLD activity during the voluntary selection condition in healthy subjects [fixed effects analysis; *q*(FDR) < 0.05]**. Increased BOLD responses are demonstrated especially in medial and lateral–frontal areas as well as the cuneus.

**Figure 5:**
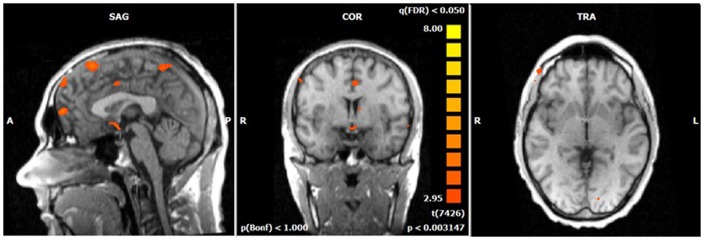
**N2-related BOLD activity during the voluntary selection condition in ADHD patients [fixed effects analysis; *q*(FDR) < 0.05]**. Increased BOLD responses are demonstrated in the same brain network as in healthy subjects including medial and lateral–frontal areas as well as the cuneus.

**Figure 6:**
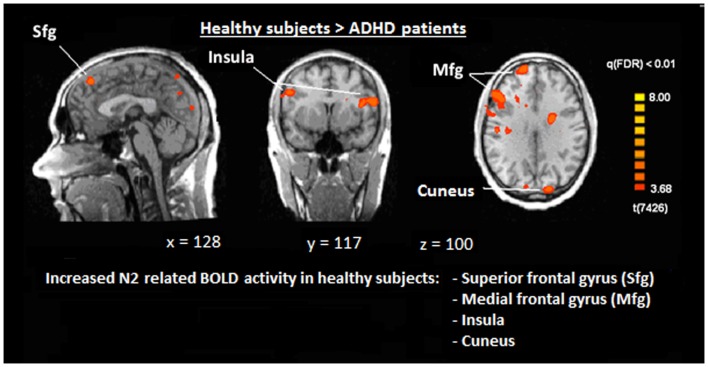
**Comparison of N2-associated BOLD activity of healthy subjects and ADHD patients during voluntary selection condition [fixed effects analysis; *q*(FDR) < 0.01]**. Healthy subjects demonstrated increased responses, e.g., in the superior frontal gyrus, medial-frontal gyrus, insula, and cuneus.

**Figure 7:**
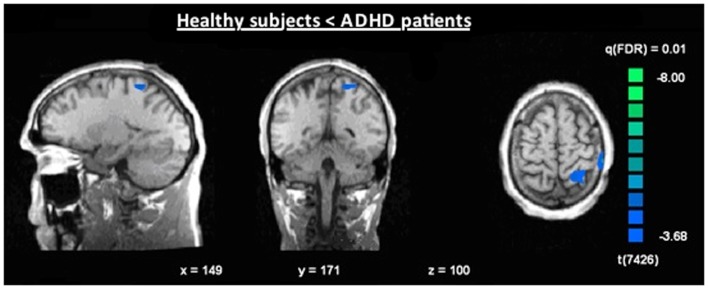
**Increased N2-associated BOLD activity of ADHD patients compared to healthy controls during voluntary selection condition [fixed effects analysis; *q*(FDR) < 0.01], e.g., in the superior parietal cortex**.

The single-trial analysis for patients and healthy controls revealed N2-associated responses pre-dominantly in the medial and lateral-frontal brain regions, including the superior and the medial-frontal gyrus (BA 6/8/46), the dorsolateral pre-frontal gyrus and the pre-central gyrus. Parietal and occipital regions, e.g., post-central gyrus (BA 3), caudate nucleus (BA 31), posterior cingulate gyrus (BA 29), and cuneus (BA 7/18/19) were involved to a lesser extent [see Figures 4 and 5; *q*(FDR) < 0.01; *T* = 3.68–8; cluster size >30 Voxel]. These results indicate that a comparable network of brain regions appears to be associated with the N2 in patients and controls. ADHD patients, however, showed reduced BOLD responses especially in frontal brain regions especially in the superior frontal gyrus (BA 8/10), the medial-frontal gyrus (BA 6/8/9), and the middle frontal gyrus (BA 6/8/9) during voluntary responses compared to healthy subjects. In addition, N2-related posterior brain responses in the cuneus (BA 7/18/19), the middle occipital gyrus (BA 19), and the limbic lobe (BA 1, 31) were less pronounced in ADHD patients. By contrast, brain responses in parietal brain regions did not differ between groups (see Table 3; Figure 6). BOLD responses were increased for ADHD patients compared to healthy subjects in the left superior parietal lobe (BA 5; L) and the post-centralis gyrus (BA 2; L) (see Table 4; Figure 7).

**Table 3:** **Single-trial analyses: N2-related BOLD activity; areas that showed higher BOLD activity in healthy subjects compared to ADHD patients during voluntary selection tasks [*q*(FDR) < 0.01; *T* = 3.68–8; cluster size >30 Voxel]**.

BOLD response during voluntary selection: healthy subjects > ADHD patients: *q*(FDR) < 0.01								
Cerebral region	BA	Side	Ø *T*-score	max. *T*-score	Size	Center of mass		
						*x*	*y*	*z*
**FRONTAL LOBE**								
Superior frontal gyrus	8	L/R	4.11	4.95	934	−1	41	48
	8	R	4.04	4.83	913	16	28	45
	10	R	4.13	5.15	935	17	61	27
Medial-frontal gyrus		L/R	4.07	5.27	5314	−38	23	22
	6	L	3.91	4.33	230	−22	−14	52
	8	R	3.94	4.62	410	32	17	46
	9	R	4.01	4.80	2644	49	19	30
Inferior frontal gyrus	46	R	4.15	5.00	890	46	37	8
Pre-central gyrus	6	R	3.85	4.19	184	34	−14	29
**LIMBIC LOBE**								
Insula	13	R	4.10	4.91	377	30	−36	16
	13	L	3.88	4.38	712	−41	−8	13
Posterior cingulate gyrus	31	L	3.88	4.38	223	−17	−61	16
Nucleus caudatus		L	4.07	4.85	923	−20	2	28
**PARIETAL LOBE**								
Post-central gyrus	3	R	3.97	4.61	325	49	−14	30
**OCCIPITAL LOBE**								
Occipital medial gyrus	19	L	3.83	4.17	238	−40	−77	11
Cuneus	7	R	3.81	4.08	95	2	−68	32
	18	R	4.02	4.86	930	7	−84	18
	19	L	4.05	4.85	737	−15	−87	29

*BA, Brodmann’s area; R, right hemisphere; L, left hemisphere; Ø *T*-score, average *T*-score of the particular cluster; max. *T*-score, maximal *T*-score of the particular clusters; size, number of activated voxels; center of mass indicated in Talairach-coordinates*.

**Table 4:** **Single.-trial analyses: N2-related BOLD activity; areas that showed less BOLD responses in healthy subjects compared to ADHD patients during voluntary selection tasks [*q*(FDR) < 0.01; *T* = 3.68–8; cluster size >30 Voxel]**.

BOLD response during voluntary selection: healthy subjects < ADHD patients: *q*(FDR) < 0.01								
Cerebral region	BA	Side	Ø *T*-score	max. *T*-score	Size	Center of mass		
						*x*	*y*	*z*
**PARIETAL LOBE**								
Superior parietal gyrus	5	L	−4.17	−5.06	737	−23	−44	58
Post-central gyrus	2	L	−4.23	−5.25	418	−50	−26	58

The P3-related BOLD responses did not differ significantly between patients and controls.

The ROI analysis demonstrated significantly decreased BOLD responses in ADHD patients in the right and left DLPFC, medial-frontal cortex, medial-posterior cortex, and right pre-frontal cortex. Neuronal activity in the parieto-occipital ROI did not differ between groups (see Table 5).

**Table 5:** **Differences between healthy subjects’ and ADHD patients’ BOLD activity in the different ROIs**.

ROI	Healthy subjects		ADHD patients		*p*-Value
	Ø size	SD	Ø size	SD	
DLPFC right	13430.25	11077.65	3259.13	5340.70	0.010
DLPFC left	5279.25	3925.69	1567.14	2224.04	0.040
Medial-frontal	3702.13	2921.482	1096.50	1754.50	0.038
Medial-posterior	1031.25	1120.38	40.50	52.33	0.013
Pre-central right	576.29	480.49	13.67	8.50	0.017
Not significant
Parietal occipital	742.40	714.85	464.00		1.000

## Discussion

The aim of the present study was to improve the understanding of the temporal characteristics of neuronal processes underlying cognitive dysfunctions in ADHD patients. The integration of a single-trial analysis allowed a functional and temporal dissociation of brain functions. With the aid of the single-trial analysis, it is possible to distinguish between neuronal responses that are associated with relatively early, N2-associated cognitive processes and later, P3-associated processes.

The results of the present study demonstrated that in both groups pre-dominantly frontal areas including the superior and medial pre-frontal gyrus, the dorsolateral pre-frontal cortex, and the pre-frontal gyrus, were related to earlier processes during the voluntary selection process. Parietal regions, the cuneus, the posterior part of the cingulate cortex, and the caudate nucleus demonstrated to be associated to a lesser extent.

These results are in line with those of previous studies: frontal areas as well as parietal areas have been related to voluntary actions, conflict detection, and decision-making processes in several previous studies [e.g., Ref. (7, 8, 13, 18, 60, 61)]. Studies about the voluntary behavior demonstrated the importance of medial-frontal responses [e.g., Ref. (1, 8, 13, 18, 23)]. Apart from frontal responses other regions have demonstrated to be relevant for intentional behavior and the generation of movements including the inferior parietal gyrus [see also Ref. (8, 18)].

Concerning the electrophysiological data, some evidence exists that the N2 is related to the suppression of motoric responses (62). In addition, pronounced N2-potentials have also been seen in the context of various executive functions, e.g., voluntary behavioral responses, intentional actions, suppression of inappropriate responses, and during response selection (13, 18, 21, 35, 62–65).

Inhibitory responses and selection processes are also related to fronto-centrally located P3 potentials (13, 28, 35). The P3 has also been linked to selective attention (66) and response preparation as well as target detection (67).

In the present study, we were able to replicate the fronto-central localization of the N2 and P3 during response inhibitions as well as voluntary selection task. However, we did not find any significant differences between ADHD patients and controls. P3-related differences between ADHD patients and healthy subjects have been demonstrated before (39). Reason for these unexpected results might be the relatively small sample size and a great heterogeneity between patients.

A function distinction between N2 and P3-related brain responses has been shown: N2-related hemodynamic responses were seen especially in medial and lateral–frontal brain regions whereas the P3 amplitude proved to be pre-dominantly related to increased BOLD responses in the temporo-parietal junction and lateral–frontal brain regions (13). These results provided some evidence that frontal brain regions are involved at an earlier stage than temporo-parietal regions, probably indicating a top-down process (13).

The direct comparison of N2-related brain responses of patients and healthy subjects demonstrated decreased BOLD responses in the medial and lateral pre-frontal areas [e.g., inferior frontal gyrus (BA 8, 10), medial-frontal gyrus (BA 6, 8, 9), inferior frontal gyrus (BA 46) as well as the pre-central gyrus (BA 6)]. Apart from the differences in frontal areas, smaller differences were also demonstrated in the caudate nucleus, in the occipital cortex (e.g., cuneus, medial occipital gyrus), and in the insula. By contrast, ADHD patients showed enhanced neural responses compared to the control group in the superior parietal gyrus (BA 5) and post-central gyrus (BA 2).

We did not find any differences regarding the P3-associated responses between ADHD patients and controls. This result may indicate that the activity of P3-related brain areas is comparable in both groups.

Neural dysfunctions in frontal, especially medial and lateral pre-frontal, areas during executive tasks (15–17) including voluntary selection processes (35) have been seen before. The present results, however, indicated that functional variations in ADHD patients that are associated with the voluntary selection between response alternatives are linked to relatively early aspects of processing whereas later aspects of task processing demonstrated to be not affected. These early aspects of task-related neuronal responses might indicate top-down processes that influence later stages of information processing.

In summary, a widely ramified network of brain areas seems to be related to decision-making procedures in healthy adults and ADHD patients including medial- and lateral-frontal brain regions, e.g., DLPFC and ACC as well as parietal and occipital areas. These findings are in line with those of previous studies with healthy subjects (1, 8–12, 68). The results are supported by the fact that, in fact, some cerebral regions are particularly altered in ADHD patients, especially medial-frontal areas. In ADHD patients pre-dominantly early aspects of decision-making were affected, whereas later aspects seem to be unaffected during voluntary tasks. Otherwise, these findings offer interesting insights into the basis of decision-making processes indicating how early a decision is drawn and occasioned.

### Limitations

The sample size of the study was relatively small (eight patients and eight healthy subjects). For that reason, a fixed effects analysis was used to calculate differences between groups. Fixed effects analyses provide only a limited possibility of generalization of the results. However, all ADHD patients were not medicated and were carefully assessed regarding their present and former ADHD symptomatology. In addition, the matching between patients and controls was done were precisely taking into account several different aspects (e.g., age, gender, intelligence level, and years of education). Nonetheless, these results should be considered preliminary.

## Conclusion

The results of the present study provide some additional knowledge about the temporo-spatial structure of deficits in ADHD patients. This information contributes to a better understanding of neurobiological processes regarding executive functions and especially voluntary actions in ADHD patients. The results provide some evidence for disturbed processes during early aspects of information processing that are located in frontal areas and result in disturbed decision-making processes. In general, the single-trial coupling of data sets seems to be a useful method in order to improve the simultaneous EEG and fMRI imaging with its high spatiotemporal resolution (39, 46). It is a promising approach to gain more insight and to understand the background and fundamental factors of several further psychiatric and neurological research questions and disease patterns.

## Conflict of Interest Statement

All authors have agreed to the submission of this article in this form. The authors reported no biomedical financial interests or potential conflicts of interest. They declare that the research was conducted in the absence of any commercial or financial relationships that could be construed as a potential conflict of interest.
